# Frequency of Toxoplasma and *Toxocara* Sp. Antibodies in Epileptic Patients, in South Western Iran

**Published:** 2015

**Authors:** Sudabeh ALLAHDIN, Shahram KHADEMVATAN, Abdollah RAFIEI, Aliakbar MOMEN, Reza RAFIEI

**Affiliations:** 1 Cellular and Molecular Research Center & Dept. Parasitology, Ahvaz Jundishapur University of Medical Sciences, Ahvaz, Iran; 2 Department of Medical Parasitology and Mycology & Cellular and Molecular Research Center, Urmia University of Medical Sciences, Urmia, Iran; 3 Cellular and Molecular Research Center, Ahvaz Jundishapur University of Medical Sciences, Ahvaz, Iran; 4 Dept. of Medical Parasitology &Infectious and Tropical Diseases Research Center, Health Research Institute, Ahvaz Jundishapur University of Medical Sciences, Ahvaz, Iran; 5 Dept. of Pediatrics, Neurology Ward, Golestan Teaching Hospital, Ahvaz Jundishapur University of Medical Sciences, Ahvaz, Iran

**Keywords:** Epilepsy, *Toxoplasma*, *Toxocara*, Seroprevalence

## Abstract

**Objective:**

Epilepsy is a disorder of the brain characterized by an enduring predisposition to generate seizures. Infectious agents are mentioned in its etiology. With identifying and appropriate treatment of these infectious agents, preventing their secondary outcomes, including seizure is possible. This study was conducted to determine frequency of anti-*Toxoplasma *antibodies (IgG, IgM) and anti-*Toxocara *antibody (IgG) in epileptic patients.

**Materials & Methods:**

Study sample consisted of 141 epileptic patients and 144 healthy people. After obtaining informed consents and completing demographic questionnaire, serum samples were taken from participants. The diagnostic test of *Toxoplasma *IgG & IgM and *Toxocara *antibodies was performed under the same conditions using ELISA method in a qualified private laboratory. Samples from patients and control groups with positive ELISA test in terms of anti-*Toxocara *antibody were also used for confirmatory Western blot test.

**Result:**

According to ELISA results, 28 (19.85%) epileptic patients and 2(1.38%) of healthy people had anti-*Toxocara *antibodies (P<001), while 39 (30.46%) of the control group people and 14.18% of patients had anti-Toxoplsma antibodies (P=0.001).

**Conclusion:**

Frequency of anti-*Toxoplasma gondii *is lower in epileptic than healthy individuals and this result is contrary to investigations that have reported higher levels of this antibody in such patient groups. ELISA results for *Toxocara *showed that the frequency of anti-*Toxocara *antibody in epileptic patients might empower the probability that this parasite may cause central nervous system damage. Western blotting has high specificity and is a proper confirmative method for diagnosis of toxocariasis.

## Introduction

“Epilepsy is a disorder of the brain characterized by an enduring predisposition to generate seizures and by the neurobiologic, cognitive, psychological and social consequences of this condition”(1). Clinical presentations are caused by different attacks, and depend on which part of the brain is impaired (2, 3).

Burden of the disease is estimated at 0.5%, and of 50 million people worldwide that suffer from this disease, more than 80% have not been cured (4). 

Infectious agents are mentioned in its etiology. 

Identifying these agents and preventing their secondary outcomes, including seizure, is an important step forward. In examining infectious agents, studies indicate the role of some helminths and protozoa such as *Taenia sollium, Toxoplasma*, *Toxocara *sp., and *Plasmodium *in causing epileptic attacks (5, 6).

*Toxoplasma gondii *infection is one of the most common parasitic infections in humans and other warm-blooded animals with worldwide distribution.

*T. gondii *is compulsive intracellular protozoa and asymptomatic in latent form in healthy people, and in immunocompromised patients, cerebral toxoplasmosis can cause seizures and intracranial mass lesions (7).

The latent form of *T. gondii *is a common and dormant infection in the brain and has the potential to cause epilepsy (8).

Recently, neuropathophysiological studies indicate a relationship between formation of microglial nodule in *Toxoplasma *infection and epilepsy. This finding increases the likelihood of infection as a cause of epilepsy (9).

Another parasitic infection that increases likelihood of epilepsy is *Toxocara*, a nematode worm of acaridae family that exists in small intestines of their natural hosts, including cats and dogs. Eggs containing second-stage larvae are accidentally ingested by humans, and hatch in the intestine, and pierce intestinal wall and migrate to various parts of the body through blood circulation (10). Since *Toxocara *sp. unable to mature under normal conditions in humans, the second-stage larvae create granulomatous inflammatory foci in these tissues after entering various body tissues and stimulation of the immune system (11).

*Toxocara *sp. may attack host’s brain and cause cerebral toxocariasis. Brain damage is caused by biological, physical, and chemical factors. It can lead to disability and even death. Since different brain damages have different results, identifying factors involved in brain damage helps determining pathophysiological mechanisms and predict neurological outcomes (12).

*Toxocara *can increase symptoms of seizure and epilepsy (13), while this parasite is not involved in pathogenesis of epilepsy, and merely there is a high prevalence of this parasite in epileptic patients (14).

*Serological techniques are the main methods in identifying Toxoplasma *and ELISA is the most common serology test to identify *Toxocara *sp, which uses excretory/secretory (E-S) parasitic antigens. It seems, under unfavorable conditions, antigens E-S allows *Toxocara *to escape the host immunity and survive. Besides, due to potential cross-reaction with other intestinal nematodes such as Ascaris, Western blot confirmatory test is used, which is also based on parasitic antigens E-S reaction with antibodies in suspect person’s serum after a certain time (15, 16).

In Iran, a high prevalence of *Toxoplasma *infection has been reported in various groups (7, 17). Toxocariasis is a helminth zoonotic infection, which is preventable (18).

Furthermore, there are contradictory results about the role of *Toxocara *and *Toxoplasma *in etiology of epilepsy (13, 14).

Therefore, this study was conducted to determine the frequency of serum anti-*Toxoplasma *specific antibodies (IgG, IgM) and anti-*Toxocara *specific antibody (IgG) in epileptic patients and comparing them with healthy people as control groups.

## Materials & Methods

**Sampling**

Study sample consisted of 141 patients presenting to neurology clinic (Dept. of Pediatrics, Neurology Ward, Ahvaz Jundishapur University of Medical Sciences, Ahvaz, Iran), with psychiatric records at health center and diagnosis of epilepsy whose electroencephalography (EEG), brain CT scan or MRI had been reviewed by neurologist in the Neurology Department of the Jundishapur University of Medical Sciences in 2014, and 144 healthy people from general population without a history of previous seizures or their families (as the control group). The types of seizure were defined according to the International League against Epilepsy (ILAE) 2001 classification (19). We selected 117 patients with idiopathic seizure, 10 patients with febrile seizure (Special syndrome) and 14 patients with generalized seizure. Patient and control groups were selected using convenient sampling, and matched in terms of age, gender, and epidemiological data determining the socioepidemiological status, and living place (urban/rural).

After obtaining informed consents and completing demographic questionnaire, serum samples were taken from participants. The study was approved by the Ethical Committee of the university with code No. of Ajums. 

REC.1392.116.

Five milliliters blood was taken from patients and control group under sterile condition, transferred to a qualified private laboratory in Ahvaz metropolitan city and centrifuged at 2000 rpm for 10 min; the separated serum was kept at -20 °C for doing the serologic tests. 

Due to potential error in test results, hemolyzed samples were not used.

**Serology and Western blotting**

The diagnostic test of *Toxoplasma *antibody was performed under the same conditions using ELISA method, and all samples were examined in terms of presence and level of *anti-T. gondii *IgG and IgM.

The results were recorded by ELISA reader as optical absorbance. Quantitative examination of samples was performed by plotting standard absorbance curve with the help of optical absorbance of their positive and negative controls and their known concentrations. 

In relation to *Toxocara*, the presence of IgG antibody was investigated in patients’ samples, and results were calculated according to optical absorbance and ELISA method against optical absorbance of control cases.

Then, for confirmation, all positive results were also tested by Western blotting terms of *Toxocara*.

ELISA *Toxocara *sp. tests were performed with NOVATEC kit (GMBH, Germany) and *Toxoplasma *tests with EUROIMMUN kit (EUROIMMUN AG, Germany). According to the kit protocol, samples were first diluted at ratio of 1:101 using kit’s diluents solution, and amount of 100λ were poured into the wells. Positive and negative controls and calibration were also used. Wells were incubated at room temperature for 30 min, and then rinsed three times. Next, 100 λ of conjugate was added and incubated again at room temperature. 

Following three times rinsing, 100λ of substrate was added and incubated. Finally, 100λ of stop solution was added and samples’ OD was read at wavelengths 450 nm or 620 nm.

In Western blot method, LD BIO Diagnostic kit (France) was used. Briefly, strips were left adjacent to 1/100 diluted serum samples at room temperature for 90 min, and after minimum of three times rinsing with saline phosphate buffer containing 0.1% tween 20, strips were incubated at room temperature with anti-human immunoglobulin G antibody conjugate peroxidase, and following rinsing stages, diaminobenzidine substrate was added, and finally, reaction was stopped by several times rinsing with distilled water. Results containing bands with low molecular weight in 24-35 kDa range were considered positive (19).

## Results

Study samples consisted of 141 patients with seizure disorder and 144 healthy people. In patients’ group, 84 were male (59.57%) and 57 were female (40.43%), and the control group consisted of 68 males (47.22%) and 76 females (52.78%). Mean age of participants was 2-39 yr. Epidemiologic and demographic characteristics of both groups are presented in Table 1.

Prevalence of *Toxocara *antibody in epileptic patients was significantly higher than that in the control group (P<0.002). According to ELISA results, 28 (19.85%) patients with seizure disorder had anti-*Toxocara *antibody (Table 2). Out of those with positive serums, 18 (21.1%) were male, and 10 (17.5%) were female. Of the 144 healthy people, 2 (1.38%) had positive results. Twenty patients with seizure disorder had anti- *Toxoplasma *antibodies, while 39 out (30.46%) of the

control group people also had this antibody (Table 2).

Results are statistically significant (P=0.001). Of the 20 serum positive patients, 12 (15%) were male. 

The frequency of anti-*Toxoplasma *(IgG) and anti-*Toxocara *antibodies measured between different types of seizures subtypes. As presented in Table 3 the frequency of *Toxoplasma *and *Toxocara *antibodies were higher in idiopathic and generalized epilepsy subtypes. 

The prevalence of *T. gondii *and *Toxocara *was compared in various age subgroups in healthy and patient groups, and the difference was significant in *Toxoplasma *(P<0.001) and *Toxocara *(P<0.05) (Table 4, 5). 

Samples from patients and control groups with positive ELISA test in terms of anti-*Toxocara *antibody were also used for confirmatory Western blot test. Positive Western blot strips indicated the presence of anti-*Toxocara*-specific antibody in samples with bands with low molecular weight in 24-35 kDa range.

**Table 1:** Demographic characteristics of the patient and control groups

Feature	Frequency		
	Patients group (%)	Control group (%)	Total (%)
**Sex**			
Female Male	57(40.43) 84(59.57	76(52.78) 68(47.22)	133(46.83) 152 (53.16)
**Residence**			
Urban Rural	48(34.04) 93(65.95)	129(89.58) 15(10.42)	167(60.72) 108(39.27)
**Marital status**			
Single Married Divorced/widowed	118(83.68) 23(16.32)	141(97.92) 3(2.08)	259(90.87) 26(9.12)
**Ethnicity**			
Fars Arab Lor Other	23(16.31) 109(77.30) 9(6.38) 0(0)	57(39.58) 34(23.61) 24(16.66) 29(20.13)	80(28.07) 143(50.17) 33(11.57) 29(10.17)
**Age (yr)**			
<20 20-29 30-39	102(72.34) 35(24.82) 4(2.83)	90(62.5) 51(35.41) 3(2.08)	19(67.36) 86(30.17) 7(2.45)

**Table 2:** Seropositivity of *Toxoplasma gondii *(IgG& IgM) and *Toxocara *IgG antibodies in epileptic patients and control group

	**Patients' group**	**Healthy**			
	**n (%)**	**n (%)**	**Sig**	**OR**	**CI**_95_
T. gondii IgG	20/141 (14.18%)	39/144 (30.46%)	0.001	0.3	0.2-0.6
T. gondii IgM	12/141 (8.51%)	0/144 (0%)	0.005	0.9	0.87-0.96
Toxocara sp	28/141 (19.85%)	2/144 (1.38%)	< 0.001	17.59	4.1 -75.42

**Table 3:** The distribution of *Toxoplasma gondii *(IgG)and *Toxocara *antibodies in patients with different subtypes of epilepsy

**Subtypes of epilepsy**	**T. gondii** **n ** **(%)**	**𝑃** **value**	**Toxocara** **n ** **(%)**	**𝑃** **value**
Idiopathic	16/117 (13.67)	0.8	25/117(21.36)	0.2
Febrile seizure(Special syndrome)	2/10 (20)		0/10(0)	
Generalized	2/14 (14.28)		3/14 (21.42)	

**Table 4:** The distribution of toxoplasmosis according to the age in epileptic patients and healthy control group

	**Patients group**	**Healthy**			
	**n (%)**	**n (%)**	**Sig**	**OR**	**CI**_95_
Age (yr)					
<20	19/102(18.62 %)	21/90(23.07%)	0.3	0.77	0.37-1.5
20-29	1/35 (2.85 %)	21/51 (42.22%)	<0.001	.04	0.005 -0.31
30-39	0/4 (0)	2/3(66.66%	0.1	3	0.6-14.86

**Table 5:** The distribution of toxocariasis according to the age in epileptic patients and healthy control group

	**Patients group**	**Healthy**			
	**n (%)**	**n (%)**	**Sig**	**OR**	**CI**_95_
Age (yr)					
<20	23/102 (22.54%)	1/90 (1.11%)	<0.001	25.91	3.42-196.29
20-29	5/35 (14.28%)	0/51 (0)	0.009	0.85	0.74 -0.98
30-39	0/4 (0)	1/3 (33.33%)	0.4	1.5	0.67-3.33

**Table 6:** The distribution of contact with cat and soil in epileptic patients and healthy control

	**Patients with epilepsy** **n (%)**	**Healthy Controls** **n (%)**	**P. value**	**CI95**
Contact with cat	41/141 (29.07)	100/141 (70.92)	<0.001	0.23-0.61
Without Contact with cat	75/144 (52.08)	69/144 (47.91)		
Contact with soil	105/141 (74.4)	36/141 (25.53)	0.002	1.29-3.54
Without Contact with soil	83/144 (57.63)	61/144 (57.36)		

## Discussion

There is no report on anti-*Toxoplasma *or *Toxocara *antibodies between epileptic patients in Khuzestan Province. In the current investigation, measurement of such antibodies was performed considering the screening of epileptic patients and healthy persons. 

In the current investigation, frequency of anti-*Toxoplasma *antibodies in case (14.18%) was lowers than control (30.46%) individuals. To survey anti-*Toxoplasma *antibody in a serologic study conducted in Khoramabad, west of Iran, between 85 epileptic patients, and have reported 14.1% positive cases while only 4.7% of control individuals were positive. Considering case individuals, our result is in accordance with previous study conducted in west of Iran (7), while our control group had higher prevalence rate. The problem why anti-*Toxoplasma *antibody frequency is lower in epileptic persons than control individuals needs more surveying. 

One cause maybe more supports and consequently higher care levels of epileptic patients, which are done by their parents. In the mentioned work, result for control group (4.7%) was lower than current investigation (30.46%) that regarding high prevalence rate of toxoplasmosis in normal population and declaration of investigator, needs cautious interpretation (7). Such difference in results could be due to differences in life style of both regions and self-health caring. 

In the current study, the frequency of anti-*Toxocara *antibody in epileptic and control groups was 19. 85% and 1.38%, respectively. In a different study, Zibaie, et al. investigated anti-*Toxocara *antibody’s frequency in 85 epileptic patients and 85 normal individuals that according to ELISA results the prevalence rate in cases was 11.8% and in controls was 3.5% (20) that the results of patients in our study (19.85%) are above than mentioned study. However, the results of control group of Zibaie et al. and our study (1.38%) are in accordance with together. In addition, they have tested anti-*Toxocara *positive samples with western-blotting method and confirmed the presence of low molecular weight bands in the zone of 24-35 kDa as positive test.

In a study in Egypt, El-Tantawy and colleagues (21) have compared the prevalence of *Toxoplasma *and *Toxocara *in epileptic patients and control individuals. In that investigation, according to ELISA results, frequency of toxoplasmosis in patients and control groups was 60.6% and 43.3%, respectively and frequency of toxocariasis was 48.5% and 46.7% in patients and controls, respectively that such considerable difference between that study and current investigation could be due to cultural, health levels of both region and nutritional habits differences. 

The hypothesis of protozoan and helminthes parasite relationships with neurological disturbances is evaluated in various studies. Khademvatan et al. investigated the frequency of anti-*Toxoplasma *and *Toxocara *IgG antibodies in schizophrenia patients in Khuzestan Province (22). Anti-*Toxocara *antibodies were 14% and 4.3% respectively and anti-*Toxoplasma *antibodies were 34% and 47.3% respectively (22). In another study, toxoplasmosis frequency was evaluated in schizophrenia patients and the frequency of anti-*Toxoplasma *antibodies was 67.7% in patients but 37.1% in control category (23). In another study, the frequency of anti-*Toxoplasma *antibody was evaluated in bipolar disorder patients, which was 31.6% in patients and 26.5% in control individuals (24).

Soil contamination by parasite ova and oocyst is one of the probable ways that increases the risk of toxocariasis and toxoplasmosis, consequently the probability of neurologic damages. 

Previous study in Ahvaz showed high rates of soil infection in various parts of the city (46.5%) and this high rate of soil infection may accounts as a risk factor (25).

Furthermore, the rate of infected cats with *Toxocara *is reported to be high in Ahvaz City. From 140 cat’s stools, 45% were reported infected (26). Infection of public environments such as streets and valleys, parks and town cities and increased rates of infected cats with *Toxocara*, enhances the likelihood of children involvement with parasitic agents; then such factors account as a risk factor for epilepsy.

Current study showed that the frequency of anti-*Toxoplasma gondii *IgM antibody in patients was 8.5% and was meaningfully higher than control category (Table 2). One evidence, which confirms that contact with soil and cat may increase the chance of infection, is higher rate of IgM positive results in patients. However, interpretation of such finding needs more investigations. 

Furthermore, our study findings showed that 74.46% of patients had the history of soil contact; also, the history of cat contact observed in 29.07% of epileptic patients (Table 6).

One of the problematic aspects in ELISA tests, which are based on excreted antigens of *Toxocara*, is crossreactivity with intestinal nematodes such as Ascaris that for prevention of this flaw the stool examination and/ or western blotting are performed on *Toxocara *positive samples, and because of higher specificity of western blotting method, we have used it in the present study. In the current investigation, 28 cases (19.85%) of epileptic serums were positive with ELISA method and all results were confirmed in western blotting method followed by rejection of probability of infection with intestinal nematodes.

In the present study, the frequency of anti-*Toxocara *antibodies in epileptic patients was 19.85%. This result was confirmed by western blotting method and supported with previous studies (27-29). Kim et al. have investigated toxocariasis in hyper-eosinophilic patients, from their 97 serum samples, 63 cases were positive with ELISA method that after confirmative testing using western blotting 58 ELISA-positive were positive and other 5 ELISA-positive cases were negative with western blotting (30). Furthermore, 7 ELISA-negative cases were western blott-positive (30). In the current investigation, all tested samples with western blotting method showed the band with 24-35 kDa. Observed bands were mostly in the range of 24-35 kDa and a few numbers of samples had bands in the regions between 75-100 kDa that this finding was supported earlier (28, 29), although bands with high molecular weight has low sensitivity for detection of *Toxocara*.

**In conclusion, **the frequency of anti-*Toxoplasma gondii *is lower in epileptic than healthy individuals and this result is contrary to investigations that have reported higher levels of this antibody in such patient groups. ELISA results for *Toxocara *showed that the frequency of anti-*Toxocara *antibody in patients with seizure disorder might empower the probability that this parasite may cause central nervous system damage but such conclusion needs more surveys. Furthermore, our results showed that western blotting has high specificity and is a proper confirmative method for diagnosis of toxocariasis.
